# Electroencephalographic biomarkers of epilepsy development in patients with acute brain injury: a matched, parallel cohort study

**DOI:** 10.1002/acn3.50925

**Published:** 2019-10-27

**Authors:** Vineet Punia, Zachary Fitzgerald, Xiaoming Zhang, Huan Huynh, James Bena, Shannon Morrison, Christopher R. Newey, Stephen Hantus

**Affiliations:** ^1^ Epilepsy Center Neurological Institute Cleveland Clinic Cleveland OH 44106; ^2^ Department of Quantitative Health Sciences Lerner Research Institute Cleveland Clinic Cleveland OH 44106; ^3^ Neurocritical Care Neurological Institute Cleveland Clinic Cleveland OH 44106

## Abstract

**Objective:**

This study was designed to investigate if highly epileptic electroencephalogram (EEG) findings in patients with acute brain injury increase the long‐term risk of epilepsy development.

**Methods:**

Adults patients, lacking epilepsy history, with electrographic seizures or lateralized periodic discharges (LPDs) (cases) were identified and matched based on age, mental status, and etiology with the ones lacking any epileptiform activity (controls) on continuous EEG (cEEG) during hospitalization. The primary outcome of clinical seizures after hospital discharge and their antiepileptic drug (AED) status was determined using a telephonic interview. Logistic regression models using generalized estimating equations to account for the matched nature of the data were performed.

**Results:**

A total of 70 cases [16 (22.9%) “LPDs only,” 34 (48.6%) “electrographic seizure only,” and 20 (28.6%) “both”] and controls were enrolled. A total of 22 (31.4%) cases developed epilepsy after a mean follow‐up duration of 20.6 ± 5.0 months compared to three (4.3%) controls. After adjusting for cEEG indication and follow‐up duration, the odds of cases developing epilepsy were almost 15 times higher compared to the controls (OR = 14.8, 95% CI = 2.4–92.3, *P* = 0.004). This elevated risk was despite a 10 times higher likelihood of cases to be taking AEDs at the last follow‐up (OR = 10.34, 95% CI = 3.7–29, *P* < 0.001).

**Interpretation:**

Highly epileptic EEG findings in patients with acute brain injury may serve as prognostic biomarkers of epilepsy development. Although prospective studies are required to confirm our findings, it seems that with epilepsy developing in almost one‐third cases in less than 2‐year follow‐up period, such patients may potentially be ideal candidates for epilepsy prevention clinical trials.

## Introduction

One of the greatest challenges in epilepsy research is to develop the means and methods of disease prevention. Around 20% to 60% of the all the epilepsies are symptomatic in nature depending on the geographical region and case ascertainment methodology.^1^ A majority of symptomatic epilepsies develop secondary to acute brain insults.^2^ These insults converge at the molecular and cellular level to lead to epileptogenesis.^2^ The latent period between the acute insult and clinical epilepsy onset is a prime target for epilepsy prevention therapies. However, currently, we lack reliable biomarkers to predict patients at a significantly high risk of developing symptomatic epilepsy. Electrophysiological monitoring after acute brain insults [e.g., traumatic brain injury (TBI)] in animal models of epileptogenesis is providing promising results.^3^ However, such findings have remained elusive in humans.^4^

The rapid increase in the utilization of continuous electroencephalogram (cEEG) monitoring in patients suffering acute brain insults in the last decade and a half^5^ has been a paradigm shift in the clinical care of critically ill hospitalized patients. cEEG monitoring is helping in the diagnosis of nonconvulsive seizure (NCS)/status epilepticus (NCSE)^6^ and highly epileptic EEG patterns like lateralized periodic discharges (LPDs, formerly called PLEDs)^7^, ^8^, ^9^, ^10^ in an ever increasing number of patients. While clinical acute symptomatic seizures are known to increase the risk of epilepsy development, the role of these acute EEG findings remains to be determined. Although a tremendous amount of research effort has been devoted to the early diagnosis, treatment, and impact of LPDs and NCS/NCSE^11^, ^12^ in acute settings, their long‐term impact is largely unknown.

Previously, we have investigated the development of new‐onset epilepsy after patients were found to have electrographic seizures (NCS/NCSE),^13^ LPDs,^13^, ^14^, ^15^ or generalized periodic discharges (GPDs)^15^ on cEEG. In these retrospective, medical records review‐based studies, we have shown that the risk of epilepsy development is determined by the type of cEEG findings.^13^, ^14^, ^15^ Building on this work, we designed a matched, parallel cohort study with the specific aims of finding the impact of highly epileptic cEEG features – that is, electrographic seizures and LPDs – on the risk of epilepsy development after acute hospitalization. We also investigated the long‐term antiepilepsy drug (AED) use in this patient population as it may potentially impact epilepsy development.

## Methods

cEEG at our institute is a full 10–20 EEG recording system whereby patients typically undergo monitoring for at least 24 h. It is performed most commonly in, but not restricted to, intensive care unit patients for indications that include suspected nonclinical seizures/status epilepticus as the cause of altered mental status (AMS) and motor events concerning for epileptic seizures or as part of the therapeutic hypothermia protocol. cEEG is always monitored in our central monitoring unit by a rotating pool of certified EEG technicians who provide as needed/requested updates to treating teams throughout the day. All cEEGs are reviewed once in a 24‐h period by a staff physician, who generates the final daily report using our in‐house EEG reporting system, which is also an indexed and searchable database of all the EEGs performed in our healthcare system. Every patient found to have seizures on cEEG is generally required to have at least one 24‐h session of seizure‐free period before cEEG discontinuation.

**Figure 1:**
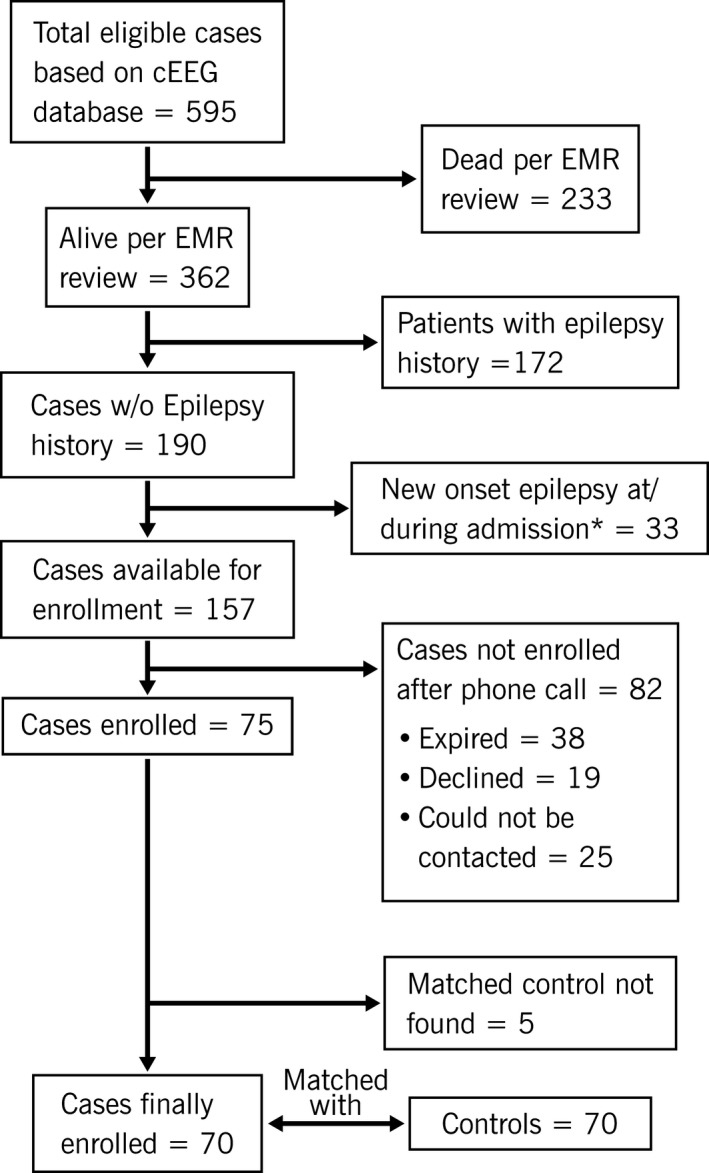
Study population flow chart. ^ = electronic medical records. *Clinical or electrographic seizure in patients with remote or progressive brain lesion.

After IRB approval, we used our prospectively maintained cEEG database from 1 January 2015 to 31 July 2016 to identify adult (≥18 years) patients who either had electrographic seizures (defined based on Salzburg criteria^16^), LPDs, or both on their cEEG (defined as “cases”). Their electronic health records (EHRs) were reviewed and following patients were excluded: passed away as per the EHR, history of epilepsy at the time of admission, diagnosed with epilepsy during hospitalization (e.g., patient with a brain tumor or old cerebral infarct presenting with clinical seizures or found to have an electrographic seizure. These patients can be diagnosed with epilepsy prior to hospital discharge based on the practical definition of epilepsy proposed by ILAE^18^). All eligible cases were contacted via mailing of research participation letters. This was followed by a telephone call‐based brief questionnaire (see below), conducted by two research assistants (ZF, XZ) blinded to cEEG findings or their group status (cases vs. controls). In situations where the patient was not able to provide answers due to language or health disability, we relied on the responses of primary caregiver. A total of three attempts, separated by a week, were made for each patient. We used cEEG database to find matched “controls”. Since the final enrolled cases determined the matched controls, the matching and identification of controls were only performed after the telephonic interviews with all potential cases were completed (Fig. 1). Controls underwent cEEG during the same study period and lacked electrographic seizures and potentially epileptic [LPDs, sporadic epileptiform discharges (sharp waves, spikes, polyspikes^17^), lateralized rhythmic delta activity (LRDA), generalized periodic discharges (GPDs)] findings. The controls were matched with cases based on age (±5 years), etiology, and mental status at the time of the beginning of cEEG.

### Study variables

Underlying etiology associated with cEEG findings was classified into three primary categories: Brain insult (primary brain pathology), anoxic brain insult, and toxic/metabolic/infectious encephalopathy (T/M/I encephalopathy – diagnosed when reversal of such etiology led to improvement in AMS). Brain insults were further categorized into: acute (within preceding 7 days of start of cEEG monitoring, for example, acute ischemic stroke, intraparenchymal hemorrhage (IPH) etc.), remote (patients with remote etiology presenting due to clinical, or found to have electrographic seizures were considered newly diagnosed epilepsy and were excluded), and progressive (e.g., tumors). Patients who had concomitant T/M/I encephalopathy along with acute/remote brain insult were categorized in the latter category.

As described above, indications for performing cEEG were classified as AMS, clinical seizure or seizure‐like events (paroxysmal, mostly motor, events like myoclonic jerks or transient unilateral posturing in comatose patients; labeled as “Clinical Sz/Sz like event” in results section), or as part of hypothermia protocol among patients who suffered cardiac arrest. Patients with AMS after a clinical seizure were categorized under “Clinical Sz/Sz like event” category.

### Telephonic questionnaire

Both cases and controls were asked two questions during a phone interview: Have you had any seizures since index (individualized for each patient) hospital admission? Are you on any antiepileptic medications (a list of AEDs was narrated upon request/if the patient was unsure)? The response to these two questions was categorized as a binary outcome of “Yes” or “No”. Patients with a response reflecting uncertainty to either question had the respective outcome labeled negative (as a “No”). Patients responding “Yes” to the first question were diagnosed with new‐onset‐epilepsy,^18^ which was the primary outcome of the study. AED status at the last follow‐up was determined based on response to the second question.

### Statistical analysis

Categorical variables were described using frequencies and percentages, while continuous variables were described using means and standard deviations, after confirming that differences followed a normal distribution graphically and using a Shapiro–Wilk test. Paired *t*‐tests were calculated for continuous variables, while McNemar's tests were used for binary factors and Stuart–Maxwell marginal homogeneity tests were calculated for multilevel categorical variables to evaluate the difference between paired cases and controls on demographic factors. For nonparametric variables, median and interquartile ranges (IQR; 1st and 3rd quartiles) were calculated and Mann–Whitney *U* test was used to compare the two groups on such variable. Logistic regression models for “New‐Onset Epilepsy” and AED at follow‐up were performed, both unadjusted and adjusting for variables that were significant in paired tests. These models used generalized estimating equations to account for the matched nature of the data. Among cases, new‐onset epilepsy and AED follow‐up were compared across findings groups (electrographic seizures only, LPDs only, and “both”) using Pearson Chi‐square tests. Analyses were performed using SAS Software (version 9.4; Cary, NC).

## Results

A flowchart of the study population (cases enrollment) is shown in Figure 1. A total of 75 cases were enrolled. Five were excluded from the analysis due to the inability to find matched controls. The remaining 70 cases were sub‐grouped based on the cEEG findings into 16 (22.9%) patients with LPDs (“LPDs only” subgroup), 34 (48.6%) patients with electrographic seizures (“electrographic seizure only” subgroup), and 20 (28.6%) patients were found to have both LPDs and electrographic seizures (“both” subgroup). The first electrographic seizure or LPDs were observed on the initial 20‐min EEG in 38 (54.3%) cases and within the first 24 h in a total of 65 (92.9%) cases. Among 54 patients with electrographic seizures, 32 (59.3%) exclusively had NCS only and the rest 16 had clinical acute symptomatic seizures (including 15 cases undergoing cEEG for indication “Clinical Sz/Sz like event”; Table 1). Combined, 48 (32 electrographic seizures + 16 “LPDs only”; 68.6%) cases did not have clinical acute symptomatic seizures.

**Table 1:** The relationship between Cases and Controls is shown below.

	Overall (*N* = 140) (%)	Control (*n* = 70) (%)	Cases (*n* = 70) (%)	*P*‐value
Mental status at cEEG onset				0.99^c^
Awake	44 (31.4)	22 (31.4)	22 (31.4)	
Coma	6 (4.3)	3 (4.3)	3 (4.3)	
Lethargy	46 (32.9)	23 (32.9)	23 (32.9)	
Stupor	44 (31.4)	22 (31.4)	22 (31.4)	
Age (years)	57.9 ± 16.1	57.9 ± 16.2	58.0 ± 16.2	0.77^a^
Gender				0.74^b^
Female	82 (58.6)	40 (57.1)	42 (60.0)	
Male	58 (41.4)	30 (42.9)	28 (40.0)	
cEEG Indication				***0.003^c^***
Altered mental status (AMS)	93 (66.4)	54 (77.1)	39 (55.7)	
Clinical Sz/Sz like event	46 (32.9)	15 (21.4)	31 (44.3)	
Hypothermia	1 (0.71)	1 (1.4)	0 (0.0)	
AMS	93 (66.4)	54 (77.1)	39 (55.7)	***0.005^b^***
Discharged on AED				***<0.001^b^***
No	64 (45.7)	59 (84.3)	5 (7.1)	
Yes	76 (54.3)	11 (15.7)	65 (92.9)	
Duration of hospitalization (days) (Median, IQR)	13 (7–21)	12 (6–19)	14 (7–23)	0.16^d^
Etiology				0.99^c^
Acute brain insult	92 (65.7)	46 (65.7)	46 (65.7)	
Stroke	26 (18.6)	13 (18.6)	13 (18.6)	
IPH	28 (20.0)	14 (20.0)	14 (20.0)	
SAH	10 (7.1)	5 (7.1)	5 (7.1)	
SDH	12 (8.6)	6 (8.6)	6 (8.6)	
PRES	6 (4.3)	3 (4.3)	3 (4.3)	
Infection	10 (7.1)	5 (7.1)	5 (7.1)	
Miscellaneous	6 (4.3)	3 (4.3)	3 (4.3)	
Progressive Brain Insult	8 (5.7)	4 (5.7)	4 (5.7)	
T/M/I Encephalopathy	34 (24.3)	17 (24.3)	17 (24.3)	
Follow‐up duration (months)	25.3 ± 6.8	30.0 ± 4.8	20.6 ± 5.0	***<0.001^a^***

IPH, Intraparenchymal hemorrhage; SAH, subarachnoid hemorrhage; SDH, subdural hemorrhage; PRES, posterior reversible encephalopathy syndrome; IQR, Interquartile range; *AMS (Yes vs. No).

Statistics presented as Mean ± SD or *N* (column %). Bold indicates statistically significant value.

*P*‐values: a = Paired *t*‐test, b = McNemar's test, c = Marginal Homogeneity test, d = Mann–Whitney *U* test.

The cases and controls were well matched by age, mental status, and etiology (Table 1). Acute etiology (central or systemic) led to the hospitalization of 90% of the study population including 65.7% with acute brain insults (details in Table 1). Eight (5.7%) patients were found to have a brain tumor (none of them had an electrographic seizure on cEEG or clinical seizure prior to the presentation). There were two elderly (>80 years of age) patients where no specific etiology could be diagnosed and one patient who suffered cardiac arrest in each group (labeled “miscellaneous” in Table 1). There was no significant difference between the two groups based on gender (*P* = 0.74). AMS was the most common indication (66.4%) for cEEG in the study population (analyzed separately in Table 1) and was a significantly more frequent reason for cEEG among the controls (*P* = 0.005). cEEG indication was included in logistic regression models by grouping the one patient with hypothermia with patients with a seizure event versus those with AMS. All, but five, cases were discharged on AEDs after cEEG monitoring compared to 15.7% of controls (*P* < 0.001) (Table 1).

### Primary outcome

**Figure 2:**
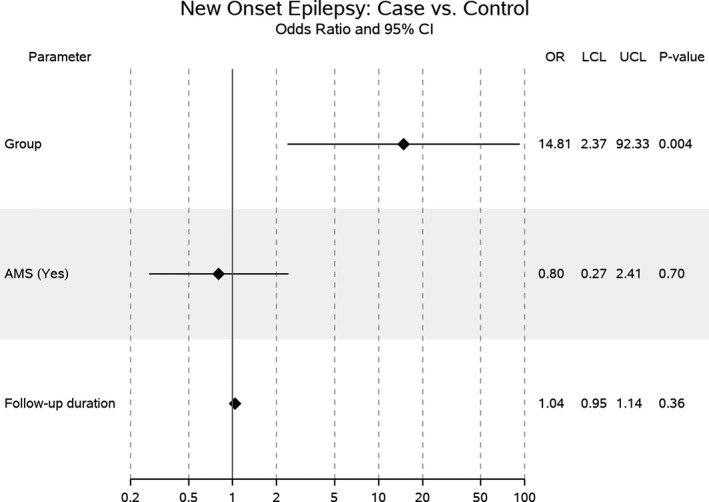
A forest plot of the multivariable model predicting new‐onset epilepsy.

A total of 22 (31.4%) cases developed new‐onset epilepsy during a mean follow‐up duration of 20.6 ± 5.0 months. In comparison, three (4.3%) controls developed epilepsy during a significantly longer duration of follow‐up of 30.0 ± 4.8 months (*P* < 0.001; Table 1). A representation of matched case‐control pairs of the study population with their mean age, mental status, and etiology are shown in Figure S1. Cases were significantly more likely [odds ratio (OR) = 10.2, 95% confidence interval (CI)  = 2.9–36, *P* < 0.001)] to develop new‐onset epilepsy compared to controls. In the multivariable model excluding AEDs at discharge, neither AMS indication nor follow‐up duration was significant predictors of new‐onset epilepsy (Fig. 2). After adjusting for indication and shorter follow‐up duration, the odds of cases developing epilepsy were further elevated compared to the controls (OR = 14.8, 95% CI = 2.4–92.3, *P* = 0.004).

### AED status at follow‐up

A total of 57 (40.7%) patients were taking at least one AED at the time of the last follow‐up. Among cases, 46 (65.7%) patients were on AED at the last follow‐up compared to 11 (15.7%) controls (OR = 10.7, 95% CI = 5.2–22, *P* < 0.001). A logistic regression model (Table 2) showed that neither indication (AMS) nor follow‐up duration were significant predictors of AED use at last follow‐up in a multivariable setting. The higher likelihood of cases to be using AEDs remained significant after adjustment of other factors (OR = 11.4, 95% CI = 4–32.8, *P* < 0.001).

**Table 2:** A logistic regression model to predict AED at follow‐up is shown below (*N* = 140).

Label	Univariable results		Multivariable results	
	Odds ratio (95% CI)	*P‐*value	Odds ratio (95% CI)	*P*‐value
AED at follow‐up: Case vs. Control	10.28 (5.03, 21.03)	***<0.001***	10.34 (3.69, 28.99)	***<0.001***
AMS (Yes)	0.31 (0.15, 0.64)	***0.001***	0.43 (0.17, 1.06)	0.066
Follow‐up duration	0.89 (0.84, 0.94)	***<0.001***	1.01 (0.93, 1.09)	0.78

Bold indicates statistically significant value.

### Cases subgroup analysis:

The primary outcome and AED status at follow‐up were separately analyzed based on cEEG subgroups among cases (Table 3). New‐onset epilepsy was not significantly different (*P* = 0.98) among patients who had electrographic seizures only, LPDs only, or both on cEEG (32.4%, 31.3%, and 30%, respectively). Similarly, there was no significant difference in being on AED among the three subgroups at the time of last follow‐up (*P* = 0.49).

**Table 3:** Distribution of primary and secondary outcomes among the controls and cases subgroups.

		Control (*N* = 70)	Cases (*N* = 70)	Electrographic seizure only (*n* = 34)	LPDs only (*n* = 16)	Electrographic seizure + LPDs (*n* = 20)	*P*‐value^*^
New‐onset epilepsy	Yes	3 (4.3)	22 (31.4)	11 (32.4)	5 (31.3)	6 (30.0)	0.98^c^
	No	67 (95.7)	48 (68.6)	23 (67.6)	11 (68.8)	14 (70.0)	
AEDs at follow‐up	Yes	11 (5.7)	46 (65.7)	22 (64.7)	9 (56.3)	15 (75)	0.49^c^
	No	59 (84.3)	24 (34.3)	12 (35.3)	7 (43.8)	5 (25.0)	

Statistics presented as *N* (column %).

*P*‐values: c = Pearson's Chi‐square test, * for cases subgroup comparison only.

### Etiologies and primary outcome

**Figure 3:**
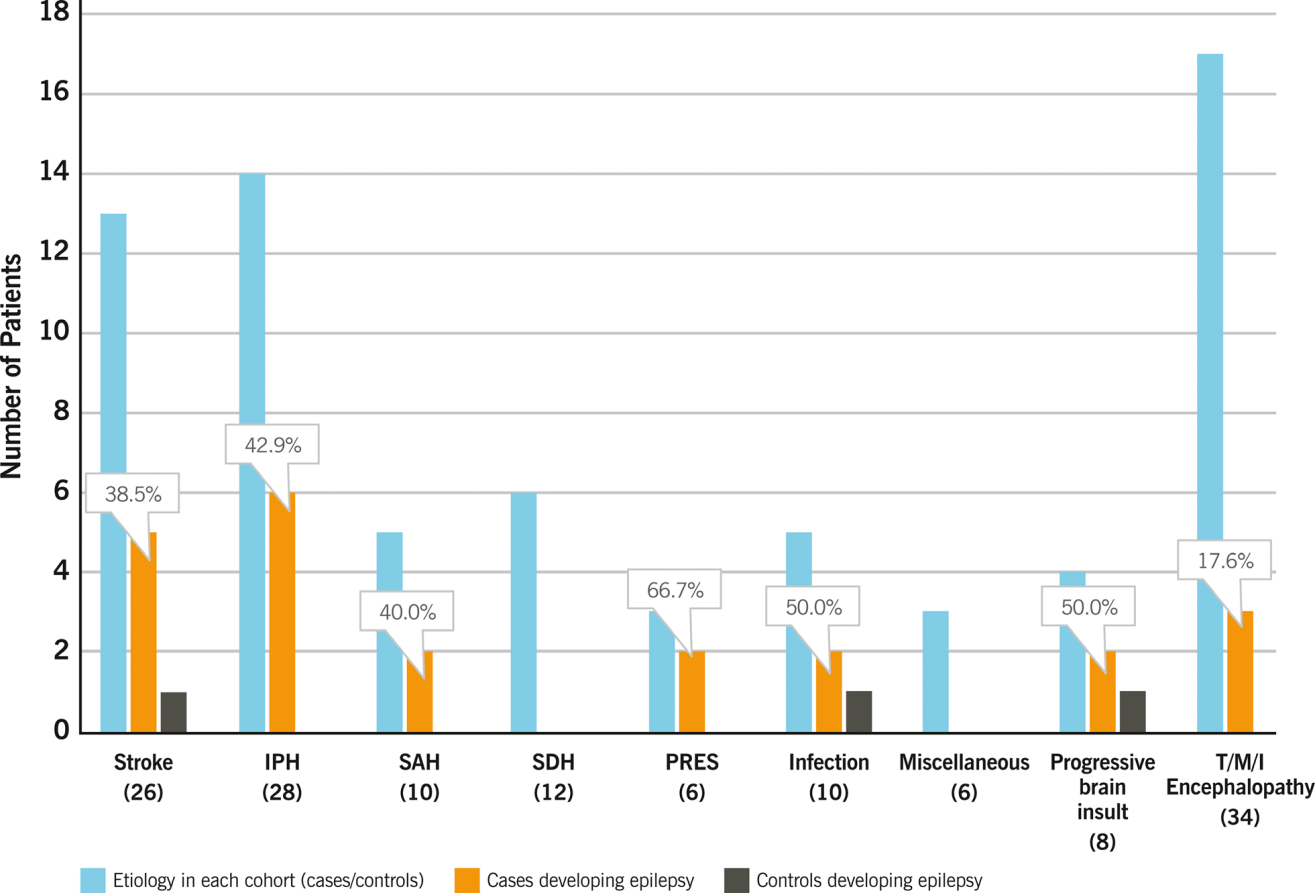
Incidence of new‐onset epilepsy among different etiologies. IPH, Intraparenchymal hemorrhage; SAH, subarachnoid hemorrhage; SDH, subdural hemorrhage; PRES, posterior reversible encephalopathy syndrome. Numbers in parenthesis represent the number of study patients in individual etiology group.

Figure 3 shows the distribution of primary outcome based on individual etiologies. Among cases, 38.5% of patients with ischemic stroke, 42.9% of patients with IPH, 40% of patients with subarachnoid hemorrhage (SAH), and 17.7% of patients with T/M/I encephalopathy developed epilepsy. Among control, only one patient each with ischemic stroke, CNS infection, and progressive brain insult developed epilepsy. None of the patients with subdural hemorrhage (SDH) and miscellaneous etiology met the primary outcome in our study.

## Discussion

Our study shows that patients of similar age, mental status during hospitalization, and etiologies may have a remarkably different risk of epilepsy development depending on the presence of certain EEG findings at the time of acute brain/systemic insult. After adjustment of co‐variables, the odds of developing epilepsy were almost 15 times higher in patients found to have LPDs or electrographic seizures on cEEG, during an average follow‐up period of 21 months, compared to patients lacking such electroencephalographic features.

The remarkably high incidence of almost one‐third of cases developing epilepsy in our study was despite 93% of these patients being discharged on AEDs. Although they had matched etiologies, the difference in AEDs at discharge between cases and controls is obviously secondary to the acutely epileptic cEEG findings among the former. However, this high frequency of AED use among patients undergoing cEEG is well known. A recent study analyzing close to 5000 patients undergoing cEEG at three tertiary‐care centers reported that close to two‐third patients received AEDs during the monitoring.^19^ Apart from patients with electrographic seizure and rhythmic/periodic patterns, close to 50% of patients without either of them on cEEG were treated with AEDs as well.^19^ Another study analyzing the influence of cEEG on AED management found that eventually 75% (225/300) patients were on AEDs by the end of cEEG monitoring and all but one were discharged on AEDs.^20^ Majority of them are likely on AEDs in the absence of clear clinical indications as noted in the other study.^19^ Interestingly in our study, more than 1.5 years after being discharged on AED, two‐thirds (65.7%) of cases were still taking at least one AED. Similar trends have been noted previously as well.^13^, ^14^ AEDs status at discharge was not used in statistical modeling because it was found to be highly collinear with the group status (95% of cases were on AEDs at discharge as compared to only 16% of controls). However, it can reasonably be argued that being on AEDs should prevent the expression of clinical seizures. This, in fact, could have led to a potential underestimation of epilepsy development among the cases in our study.

Prior studies from our group have found the risk of epilepsy development to be dependent on the EEG findings at the time of hospitalization. Only four out of 73 (6%) patients with GPDs on cEEG developed epilepsy, which was comparable to patients who did not have any periodic or epileptiform findings.^15^ In contrast, 17–38% of patients with LPDs developed epilepsy in these studies^13^, ^14^, ^15^ and the risk went as high as 48.5% if there were accompanying electrographic seizures.^13^ However, the above studies suffered from several design limitations. They had a small study population,^14^ did not include a comparative patient group,^13^, ^14^ subjects were selected based on the availability of short‐term (at least 3 months) clinical follow‐up in our EHR, and the outcomes were determined by, and dependent on, clinical documentation in the EHR. Our current study overcomes the limitation of selection bias through patient outreach via telephone interview and relying on patient‐reported clinical outcomes. We diligently matched cases (patients with electrographic seizures or LPDs on cEEG) to controls (patients lacking any potentially epileptic findings on cEEG) on critical parameters of age, mental status, and etiology. We believe this helps in the easy interpretation of our findings, whereby the tremendously high risk of epilepsy development among cases can be clearly attributed to the difference in cEEG findings between the two cohorts. Multivariable modeling further showed that epilepsy development was neither influenced by the indication for cEEG monitoring nor the follow‐up duration but was exclusively dependent on whether the patient belonged to the case or control group (Fig. 2). The control population had a significantly longer follow‐up duration because of the identification of control cohort depended on first contacting all the potential cases, which were 153 in total (Fig. 1).

Few pre‐cEEG era studies, involving a small number of patients, have reported 10–58% of patients with LPDs having seizure recurrence after hospital discharge.^21^, ^22^, ^23^ However, the EEG in these studies was performed after clinical seizures. In our study, more than two‐thirds (68.6%) of acute epileptic findings were in patients without clinical acute symptomatic seizure. A more recent study aimed at retrospectively (through chart review) analyzing the prognostic value of periodic discharges (PDs) noted on 30‐min serial EEGs used a case (with PDs) – control (without PDs) design to match patients by age, gender, and etiology.^24^ Among survivors, they found that the odds of patients with PDs developing epilepsy were 3.3 times higher than control, which was not statistically significant after regression analysis.^24^ A study utilizing cEEG in pediatric population found that NCSE led to a significantly higher risk (odds ratio 13.3) of developing epilepsy.^25^ Study of posttraumatic epilepsy (PTE) after traumatic brain injury (TBI) in humans and animals^26^ has been of great research interest to explore epileptogenesis. A study comparing matched (by age and severity) post‐TBI patients, divided into two groups based on the development of PTE, found that the presence of epileptiform abnormalities on cEEG during the acute period was an independent predictor of PTE [odds ratio = 3.16 (0.99, 11.68)].^27^ However, a critical difference between our study and the literature is that the latter either lacked controls or retrospectively determined the outcome or both.

Unlike our previous studies, we did not find the incidence of new‐onset epilepsy to vary among cases based on the presence of either electrographic seizures or LPDs or both on cEEG. This is either due to the difference in study population selection and primary outcome determination in the current study or possibly because LPDs represent an ictal‐interictal phenomenon that is as epileptogenic in long term as actual electrographic seizures. We did not look into the frequency of LPDs and the relation to primary outcome due to small sample size.

Our study was not designed to investigate the impact of prophylactic AEDs (overall or a specific AED) on epilepsy development. However, the ultimate aim of identifying at‐risk patients is to be able to prevent such outcomes. Validation of epilepsy preventing therapies through clinical trials requires the enrollment of patients at a significantly high baseline risk of developing epilepsy. Risk of PTE in adults after severe TBI, the most commonly studied high‐risk population, ranges from 7.7% in 1 year to 13.3% in 5 years.^28^ Considering a mean incidence rate of 10.5%, the sample size of such population needed to detect a 50% treatment effect (alpha 0.05, power 0.8) would be 882. This large study population, requiring follow‐up for a long duration of time, makes the conduction of epilepsy prevention trials financially unviable.^29^ In comparison, the subject population selected based on the presence of electrographic seizures and LPDs on acute EEG, with the epilepsy incidence of 31% according to our findings, would only require 254 patients. In fact, our study suggests that this highly enriched study population may require a much shorter follow‐up period and the enrollment process may be faster because these EEG findings are not restricted to a single etiology. The incidence of epilepsy after stroke and hemorrhage ranges from 10%–14%.^30^, ^31^ In comparison, 40.7% of the cases and only one (3.7%) control with such etiologies developed epilepsy (Fig. 3). These numbers are significantly divergent from general population studies and suggest that using the acute EEG information may play a critical role in clinical prognostication. Along the same lines, adding the EEG data to the etiology may be helpful for guiding the length and dosage of AED therapy. However, further research is required in this direction.

Our study is limited by its dependence of primary outcome on patient's memory recall. In an alternative study design, the memory recall could be aided by interviewing for specific signs and symptoms of seizures, followed by clinic visit for patients providing positive response. This would avoid the small possibility of epilepsy misdiagnosis. The cases are derived from an eligible population that suffers acute insults associated with a high mortality rate, accounting for the large attrition rate (Fig. 1). Recent studies among patients with ischemic stroke^32^ and intracerebral hemorrhage^30^ have shown that neuroimaging findings can be used to predict the risk of epilepsy development in this population. We did not look at the neuroimaging features among patients who suffered from acute brain insults as the aim of our study was to investigate the prognostic role of electroencephalographic findings. Future studies combining acute electroencephalographic and neuroimaging findings in patients with acute brain insults may be able to predict the risk of epilepsy development with a higher precision. We did not match acute insults like ischemic strokes or hemorrhages by their location or extent. However, matching mental status helped ensure that a specific etiology impacted brain function to a similar extent among the cases and controls. By design, we did not include patients with all form of epileptiform discharges, for example, sharp waves,^17^ LRDA^33^ etc. in our cases. For the current study, we wanted to start investigating the influence of acute cEEG findings on epilepsy development by first concentrating on the most highly epileptic findings at the time of acute insult. We lack the data on the timing of clinical seizure relative to hospital discharge, which is a limitation stemming from our attempt to simplify telephonic interview and the likelihood of an unreliable precise recall about the timing of seizure by patients. Therefore, future studies designed to prospectively follow‐up patients with electrographic seizures and LPDs are required to chart the time course of epilepsy development and validate our findings.

In conclusion, our study shows that patients with electrographic seizure or LPDs on EEG at the time of acute brain/systemic insult are at a significantly increased risk of epilepsy development. Our findings suggest that these acute EEG findings may serve as a prognostic biomarker of symptomatic epilepsy development.

## Author Contribution

Following authors contributed to the conception and design of the study: VP, CRN, SH.

Following authors contributed to the acquisition and analysis of data: VP, ZF, XZ, HH, JB, SM.

Following authors contributed to the drafting a significant portion of the manuscript or figures: VP, CRN, SH.

## Conflict of Interest

All authors report no potential conflict of interest.

## Supporting information


**Figure S1.** Paired case and control plot of follow‐up with primary outcomes.

## References

[1] Banerjee PN , Filippi D , Allen HW . The descriptive epidemiology of epilepsy‐a review. Epilepsy Res 2009;85:31–45. 19369037 10.1016/j.eplepsyres.2009.03.003 PMC2696575

[2] Klein P , Dingledine R , Aronica E , et al. Commonalities in epileptogenic processes from different acute brain insults: do they translate? Epilepsia 2018;59:37–66. 29247482 10.1111/epi.13965 PMC5993212

[3] Reid AY , Bragin A , Giza CC , et al. The progression of electrophysiologic abnormalities during epileptogenesis after experimental traumatic brain injury. Epilepsia 2016;57:1558–1567. 27495360 10.1111/epi.13486 PMC5207033

[4] Pitkanen A , Engel JJ . Past and present definitions of epileptogenesis and its biomarkers. Neurotherapeutics 2014;11:231–241. 24492975 10.1007/s13311-014-0257-2 PMC3996117

[5] Ney JP , van der Goes DN , Nuwer MR , et al. Continuous and routine EEG in intensive care: utilization and outcomes, United States 2005–2009. Neurology 2013;81:2002–2008. 24186910 10.1212/01.wnl.0000436948.93399.2a PMC3854828

[6] Claassen J , Mayer SA , Kowalski RG , et al. Detection of electrographic seizures with continuous EEG monitoring in critically ill patients. Neurology 2004;62:1743–1748. 15159471 10.1212/01.wnl.0000125184.88797.62

[7] Pohlmann‐Eden B , Hoch DB , Cochius JI , Chiappa KH . Periodic lateralized epileptiform discharges–a critical review. J Clin Neurophysiol 1996;13:519–530. 8978624 10.1097/00004691-199611000-00007

[8] Fitzpatrick W , Lowry N . PLEDs: clinical correlates. Can J Neurol Sci 2007;34:443–450. 18062453

[9] Newey CR , Sahota P , Hantus S . Electrographic features of lateralized periodic discharges stratify risk in the interictal‐ictal continuum. J Clin Neurophysiol 2017;34:365–369. 28166083 10.1097/WNP.0000000000000370

[10] Newey CR , Kinzy TG , Punia V , Hantus S . Continuous electroencephalography in the critically Ill. J Clin Neurophysiol 2018;35:325–331. 29677014 10.1097/WNP.0000000000000475

[11] Struck AF , Ustun B , Ruiz AR , et al. Association of an electroencephalography‐based risk score with seizure probability in hospitalized patients. JAMA Neurol 2017;74:1419–1424. 29052706 10.1001/jamaneurol.2017.2459 PMC5822188

[12] Husain AM , Lee JW , Kolls BJ , et al. Randomized trial of lacosamide versus fosphenytoin for nonconvulsive seizures. Ann Neurol 2018;83:1174–1185. 29733464 10.1002/ana.25249 PMC6785201

[13] Punia V , Garcia CG , Hantus S . Incidence of recurrent seizures following hospital discharge in patients with LPDs (PLEDs) and nonconvulsive seizures recorded on continuous EEG in the critical care setting. Epilepsy Behav 2015;49:250–254. 26198216 10.1016/j.yebeh.2015.06.026

[14] Punia V , Vakani R , Burgess R , Hantus S . Electrographic and clinical natural history of lateralized periodic discharges. J Clin Neurophysiol 2018;35:71–76. 29099408 10.1097/WNP.0000000000000428

[15] Punia V , Bena J , Krishnan B , et al. New onset epilepsy among patients with periodic discharges on continuous electroencephalographic monitoring. Epilepsia 2018;59:1612–1620. 29974460 10.1111/epi.14509

[16] Beniczky S , Hirsch LJ , Kaplan PW , et al. Unified EEG terminology and criteria for nonconvulsive status epilepticus. Epilepsia 2013;54(Suppl 6):28–29. 10.1111/epi.12270 24001066

[17] Noachtar S , Binnie C , FAU‐Ebersole J , et al. A glossary of terms most commonly used by clinical electroencephalographers and proposal for the report form for the EEG findings. The International Federation of Clinical Neurophysiology. Electroencephalogr Clin Neurophysiol 1999;52:21–41. 10590974

[18] Fisher RS , Acevedo C , Arzimanoglou A , et al. ILAE official report: a practical clinical definition of epilepsy. Epilepsia 2014;55:475–482. 24730690 10.1111/epi.12550

[19] Alvarez V , Rodriguez Ruiz AA , LaRoche S , et al. The use and yield of continuous EEG in critically ill patients: a comparative study of three centers. Clin Neurophysiol 2017;128:570–578. 28231475 10.1016/j.clinph.2017.01.001

[20] Kilbride RD , Costello DJ , Chiappa KH . How seizure detection by continuous electroencephalographic monitoring affects the prescribing of antiepileptic medications. Arch Neurol 2009;66:723–728. 19506131 10.1001/archneurol.2009.100

[21] Walsh JM , Brenner RP . Periodic lateralized epileptiform discharges–long‐term outcome in adults. Epilepsia 1987;28:533–536. 3653057 10.1111/j.1528-1157.1987.tb03684.x

[22] Garcia‐Morales I , Garcia MT , Galan‐Davila L , et al. Periodic lateralized epileptiform discharges: etiology, clinical aspects, seizures, and evolution in 130 patients. J Clin Neurophysiol 2002;19:172–177. 11997729 10.1097/00004691-200203000-00009

[23] Schraeder PL , Singh N . Seizure disorders following periodic lateralized epileptiform discharges. Epilepsia 1980;21:647–653. 7439132 10.1111/j.1528-1157.1980.tb04318.x

[24] Pedersen GL , Rasmussen SB , Gyllenborg J , et al. Prognostic value of periodic electroencephalographic discharges for neurological patients with profound disturbances of consciousness. Clin Neurophysiol. 2013;124:44–51. 22809812 10.1016/j.clinph.2012.06.010

[25] Wagenman KL , Blake TP , Sanchez SM , et al. Electrographic status epilepticus and long‐term outcome in critically ill children. Neurology 2014;82:396–404. 24384638 10.1212/WNL.0000000000000082 PMC3917684

[26] Engel JJ . Epileptogenesis, traumatic brain injury, and biomarkers. Neurobiol Dis 2019;123:3–7. 29625256 10.1016/j.nbd.2018.04.002 PMC6170720

[27] Kim JA , Boyle EJ , Wu AC , et al. Epileptiform activity in traumatic brain injury predicts post‐traumatic epilepsy. Ann Neurol 2018;83:858–862. 29537656 10.1002/ana.25211 PMC5912971

[28] Annegers JF , Grabow JD , Groover RV , et al. Seizures after head trauma: a population study. Neurology 1980;30:683–689. 7190235 10.1212/wnl.30.7.683

[29] Engel J Jr , Pitkanen A , Loeb JA , et al. Epilepsy biomarkers. Epilepsia 2013;54(Suppl):461–469. 23909854 10.1111/epi.12299 PMC4131763

[30] Haapaniemi E , Strbian D , Rossi C , et al. The CAVE score for predicting late seizures after intracerebral hemorrhage. Stroke 2014;45:1971–1976. 24876089 10.1161/STROKEAHA.114.004686

[31] Thurman DJ , Begley CE , Carpio A , et al. The primary prevention of epilepsy: a report of the prevention task force of the international league against epilepsy. Epilepsia 2018;59:905–914. 29637551 10.1111/epi.14068 PMC7004820

[32] Galovic M , Döhler N , Erdélyi‐Canavese B , et al. Prediction of late seizures after ischaemic stroke with a novel prognostic model (the SeLECT score): a multivariable prediction model development and validation study. Lancet Neurol 2018;17:143–152. 29413315 10.1016/S1474-4422(17)30404-0

[33] Gaspard N , Manganas L , Rampal N , et al. Similarity of lateralized rhythmic delta activity to periodic lateralized epileptiform discharges in critically ill patients. JAMA Neurol 2013;70:1288–1295. 23921464 10.1001/jamaneurol.2013.3475

