# Supplementary figures and images for: Electroencephalographic biomarkers of epilepsy development in patients with acute brain injury: a matched, parallel cohort study
Source: Ann Clin Transl Neurol. 2019 Oct 27;6(11):2230–9. doi: 10.1002/acn3.50925 (PMC6856614; doi:10.1002/acn3.50925)

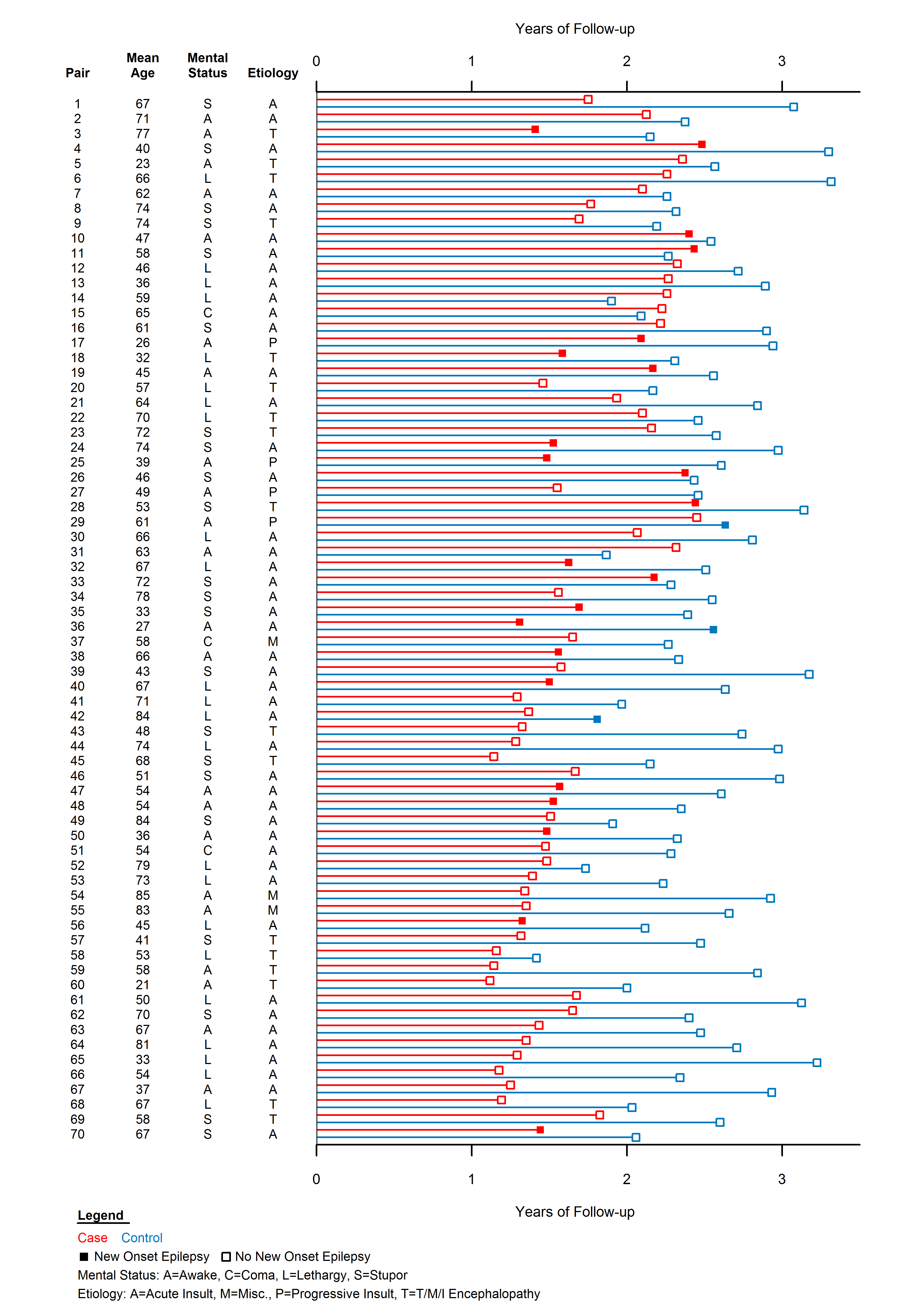

Supplement: Supplementary file 1 — Figure S1. Paired case and control plot of follow‐up with primary outcomes. [file ACN3-6-2230-s001.tif]
